# Cytotoxic Activity, Anti-Migration and In Silico Study of Black Ginger (*Kaempferia parviflora*) Extract against Breast Cancer Cell

**DOI:** 10.3390/cancers15102785

**Published:** 2023-05-17

**Authors:** Indah Hairunisa, Mohd Fadzelly Abu Bakar, Muhammad Da’i, Fazleen Izzany Abu Bakar, Eka Siswanto Syamsul

**Affiliations:** 1 Faculty of Applied Sciences and Technology, Universiti Tun Hussein Onn Malaysia (UTHM), Muar 84600, Malaysia; 2 Faculty of Pharmacy, Universitas Muhammadiyah Kalimantan Timur (UMKT), Samarinda 75124, Indonesia; 3 Faculty of Pharmacy, Universitas Muhammadiyah Surakarta (UMS), Solo 57162, Indonesia; 4 Sekolah Tinggi Ilmu Kesehatan Samarinda (STIKSAM), Samarinda 75242, Indonesia

**Keywords:** black ginger, *Kaempferia parviflora*, cytotoxic, anti-migration, metastasis, molecular docking, phytochemicals, breast cancer

## Abstract

**Simple Summary:**

Breast cancer that has metastasized to other parts of the body still has a high incidence and mortality rate. Furthermore, treating metastatic breast cancer remains a major medical undertaking. This is due to the limited treatment options for patients with metastatic breast cancer, as well as the occurrence of drug resistance when long-term treatment is used. The search for drug candidates using natural medicine is currently accelerating, particularly in Asia. Plants that have traditionally been used to improve health must be studied and scientifically proven in order to ensure their efficacy. This study demonstrates that *Kaemferia parviflora* may have the scientific potential to be developed as a cytotoxic and anti-migration agent for breast cancer. This research also predicts the bioactive compound responsible for this activity. To complete it out, this article discusses the potential mechanism of action of *Kaemferia parviflora*’s potential components. The findings of this study could pave the way for *Kaemferia parviflora* to be developed as an anticancer agent.

**Abstract:**

Metastatic breast cancer remains the leading cause of death in women worldwide. This condition necessitates extensive research to find an effective treatment, one of which is the natural medicine approach. *Kaempferia parviflora* (KP) is a plant believed to possess anticancer properties. Therefore, this study aims to determine KP’s bioactive compound, cytotoxic, and anti-migration activity in the highly metastatic breast cancer cell line model 4T1, also in the breast cancer cell model MCF-7 and noncancerous cell line NIH-3T3. Maceration with ethanol (EEKP) and infusion with distilled water (EWKP) was used for extraction. The MTT assay was used to test for cytotoxicity, and the scratch wound healing assay was used to test for the inhibition of migration. Phytochemical profiling of EEKP was performed using UHPLC-MS, and the results were studied for in silico molecular docking. Result showed that EEKP had a better cytotoxic activity than EWKP with an IC_50_ value of 128.33 µg/mL (24 h) and 115.09 µg/mL (48 h) on 4T1 cell line, and 138.43 µg/mL (24 h) and 124.81 µg/mL (48 h) on MCF-7 cell line. Meanwhile, no cytotoxic activity was observed at concentrations ranging from 3–250 µg/mL in NIH-3T3. EEKP also showed anti-migration activity in a concentration of 65 µg/mL. Mass Spectrophotometer (MS) structures from EEKP are 5-Hydroxy-7,4′-dimethoxyflavanone (HDMF), 5-Hydro-7,8,2′-trimethoxyflavanone (HTMF), Retusine, and Denbinobin. The in silico docking was investigated for receptors Bcl-2, Bcl-XL, ERK2, and FAK, as well as their activities. In silico result indicates that HTMF and denbinobin are bioactive compounds responsible for EEKP’s cytotoxic and anti-migration activity. These two compounds and standardized plant extract can be further studied as potential breast cancer treatment candidates.

## 1. Introduction

Breast cancer remains a leading cause of death in women worldwide [1]. In 2020, there are 355,457 incidents and 91,826 deaths in the EU [2]. This number is expected to rise annually. Breast cancer is responsible for 24.5% of all female cancers and half of these incidences occurred in Asia [3].

In addition, breast cancer in its advanced stages has the potential to spread, known as metastasis [4]. Cancer cells migrate and settle in distant organs, thereby forming new colonies as a result of this property. Moreover, breast cancer has a high incidence of metastasis [5]. Approximately, 15–25% of all breast cancer cases will progress to metastatic breast cancer [6]. Metastatic breast cancer is also known as the terminal stage of breast cancer.

However, breast cancer treatment can be a complicated process because of the nature of metastases. In general, systemic treatments such as hormonal therapy, chemotherapy, immunotherapy, and targeted therapy are used to treat metastatic breast cancer [7]. In addition to systemic treatment, radiation therapy and surgery are also good options [8]. However, the efficacy of this treatment remains challenging in the treatment of metastatic breast cancer [9]. These treatments initially produce positive results, but long-term and repeated treatments can result in resistance [10]. Therefore, it is necessary to seek out new and effective treatments to address this issue.

There has been an extensive search for active ingredients for cancer treatment derived from natural ingredients [11]. *Kaempferia parviflora* (KP), also known as black ginger, is a *Zingiberaceae* plant that can be potentially developed as an anticancer and antimetastatic agent. *Kaempferia parviflora* has numerous pharmacological activities that are extremely beneficial to health, including neuroprotective, antibiotic, aphrodisiac, anti-obesity, antidiabetic, anti-inflammation, and anticancer [12,13,14,15,16,17,18]. This plant is also commonly used in traditional drinks and as the primary raw material in the production of traditional medicinal preparations.

In addition, KP, in the form of crude extract and its pure compound, has a cytotoxic effect on several cancer cell models. KP inhibited the proliferation of HeLa cells [14]. It also showed antiproliferative and antimetastatic effects on a highly aggressive ovarian cancer cell SKOV3 [15]. This cytotoxic activity is thought to be caused by KP’s influence on the apoptotic pathway, in which KP induces apoptosis via the intrinsic pathway by influencing the ratio of Bcl-2, Bax, as well as Caspases 3 and 9 [16]. Based on this, KP is expected to have cytotoxic activity in breast cancer.

Moreover, KP’s potential is linked not only to its antiproliferative but also to its antimetastatic activity. Several studies on this activity have been carried out. KP demonstrated antimetastatic activity in Human bile duct cancer (HuCCA-1 and RMCCA-1), ovarian cancer cells (SKOV3), and Cervical Cancer HeLa [19,20]. These three cancers have a high proclivity to spread. In general, cancer cell metastasis is aided by the production of matrix metalloproteinases 2 and 9, which work by breaking down matrix proteins when cancer cells are about to metastasise [21]. KP demonstrated antimetastatic activity in HeLa cells and the mechanism is due to the inhibition of matrix metalloproteinase-2 production by KP [18]. Similarly, KP inhibited the activity of MMP-2 and MMP-9 at concentrations of 0.01–0.05 μg/mL in ovarian cancer cells (SKOV3) [15]. On the contrary, in human bile duct cancer (HuCCA-1 and RMCCA-1), KP could inhibit cancer cell movement when compared to controls, however, this study did not investigate the possible mechanism of KP [17]. Besides being an anticancer, KP can also be an anti-migration agent.

Based on the above-mentioned, previous research has revealed the high number of cases of metastatic breast cancer and the limited treatment options, as well as the potential of KP as an anticancer and anti-migration agent. Thus, this study aims to look into KP’s cytotoxic and anti-migration activity in the highly metastatic 4T1 breast cancer cell model, MCF-7 breast cancer model, and its safety on noncancerous cell line NIH-3T3. Furthermore, a molecular docking approach was used on the active compounds in KP against several proteins involved in the apoptotic and metastatic pathways. Furthermore, the findings of this study can be used to demonstrate KP’s cytotoxic and anti-migration activity; these two activities can then be used to evaluate KP’s antimetastatic activity.

## 2. Materials and Methods

### 2.1. Materials

This study used the rhizome of *K. parviflora*, 96% ethanol (Merck, Rahway, NJ, USA), 96% methanol PA (Merck), distilled water (Onelab, PT. Jayamas Medica Industri, Indonesia), RPMI Medium (Gibco, Billings, MT, USA), DMEM High Glucose (Gibco), MTT Reagent (Merck), Trypsin (Gibco), Fetal Bovine Serum (Gibco), sodium bicarbonate, Hepes, penicillin–streptomycin (Thermo Fischer, Waltham, MA, USA), 96-well plate (Biologic, Seyssinet-Pariset, France), micropipette (Dragonlab), rotary evaporator (RV 10), vortex (B-One, Cedar Knolls, NJ, USA), Elisa Reader (Epoch, Fremont, CA, USA), glassware (Iwaki, Tokyo, Japan), and freeze dryer.

### 2.2. Sample Preparation

The rhizome of *K. parviflora* was obtained from Johor, Malaysia. Sample authentication was carried out at the Laboratory of Ecology and Conservation of Tropical Forest Biodiversity at the University of Mulawarman, Indonesia with the herbarium number of 12/UNI7.4.08/LL/2021. The sample was cleaned using a wet cloth. Then, it was cut into small pieces for further drying in an oven at 40 °C. Subsequently, the dried sample was powdered using a blender and then sieved to obtain a sample size of less than 2 mm. Finally, samples that were uniform in size were used for extraction.

### 2.3. Sample Extraction

Samples were extracted using the following two different methods: maceration with 96% ethanol and infusion with distilled water. Maceration was carried out for 72 h at a sample-to-solvent ratio of 1:8, with stirring every 24 h. The maceration results were concentrated using a rotary evaporator to obtain a thick extract. The second extraction used an infusion technique (boiling at 90 °C for 15 min) with distilled water as a solvent [22], and the extraction results were dried using a freeze dryer.

### 2.4. Determination of Secondary Metabolites

Determination of secondary metabolites was carried out on the samples, including saponins, tannins, alkaloids, flavonoids, polyphenols, terpenoids, steroids, glycosides, and quinones. The phytochemical content was determined following standard procedures [23]. After specific reagents were added to the solution, tests were carried out by visual observation of colour change or precipitate formation.

### 2.5. Determination of Cytotoxic Activity Using MTT Assay

The MTT assay was carried out based on previous study with modifications on 4T1, MCF-7, and NIH-3T3 cell line [19]. 4T1 and MCF-7 cells are breast cancer cell models, whereas NIH-3T3 cells are noncancerous cell line models. All cell lines were kindly provided by Prof. Dr. Muhammad Dai (Cell Culture Laboratory, Faculty of Pharmacy, Universitas Muhammadiyah Surakarta, Solo, Indonesia). All the cells were grown in 96 well plates. Cell cultures were incubated for 24 h in RPMI medium (for 4T1 cell line) and DMEM (for MCF-7 and NIH-3T3 cell line) and incubated at 37 °C with a CO_2_ level of 5%. Then, 100 µL of sample (EWKP, EEKP, and Doxorubicin) solution with the concentration in the range 3–250 µg/mL was placed into the well, then incubated for 24 and 48 h. Afterward, cells were washed with 100 µL of PBS from each well, then 0.5 mg/mL of MTT reagent was added to the culture medium in each well. Incubated again for 2.5 h. Subsequently, the media containing MTT was stopped by adding a stopper reagent (10% SDS in 0.01 N HCl). The plates were wrapped in aluminum foil and left overnight at room temperature. The following day, the absorbance was read using an ELISA reader (EPOCH) with a wavelength of 595 nm. The absorbance data of a single treatment was converted into percent cell viability using this formulation:

$$\mathrm{Percent}\;\mathrm{cell}\;\mathrm{viability}\;(\%)=\frac{\left(Abs\;sample-Abs\;medium\right)}{\left(Abs\;control\;cell-Abs\;medium\right)}\times 100\%.$$

Make a linear regression equation of concentration vs. cell viability, then calculate the IC_50_ by changing the value of y = 50.

### 2.6. Determination of Anti-Migration Effect Using Scratch Wound Healing Assay

A total of 4 × 10^4^ 4T1 cells were distributed into 96 well plates, then incubated for 24 h at 37 °C, 5% CO_2_ cells were washed using PBS 1× and given starvation medium (a culture medium with 0.5% FBS) and then incubated for 24 h. Then, each well was scratched vertically using a white tip and treated with Doxorubicin 0.5 µg/mL (positive control) and EEKP 16.25 µg/mL, 32.5 µg/mL, and 65 µg/mL. Finally, cells were observed under a light microscope (Olympus, Olympus Corporation, Japan) each time at 0 h, 8 h, and 24 h and captured by the camera [19].

### 2.7. Secondary Metabolites Profiling Using Ultra-High Performance Liquid Chromatography Mass Spectrophotometry (UHPLC-MS)

Secondary metabolite studies were carried out with reference to procedure in previous study with modifications [20]. Ultra-High Performance Liquid Chromatography Mass Spectrophotometry (UHPLC-MS) was performed on the ACQUITY UPLC I-Class system from Waters. A total of 1 µL sample was injected into column ACQUITY UPLC HSS T3 (100 mm × 2.1 mm × 1.8 μm) also from Waters, and maintained at 40 °C. The mobile phase used was water and acetonitrile (0.1% formic acid) as solvents A and B, respectively. The mobile phase was flowed using a gradient technique as follows: 0 min, 1% B; 0.5 min, 1% B; 16.00 min, 35% B; 18.00 min, 100% B; and 20.00 min, 1% B. The flow rate was set to 0.6 mL/min. The ion source was operated in positive electrospray ionisation (ESI) mode under the following specific conditions: capillary voltage, 1.50 kV; reference capillary voltage, 3.00 kV; source temperature, 120 °C; desolvation gas temperature, 550 °C; desolvation gas flow, 800 L/h; and cone gas flow, 50 L/h. Nitrogen (>99.5%) was employed as desolvation and cone gas. Data were acquired in high-definition MS E (HDMS E) mode in the range of *m*/*z* 50–1500 at 0.1 s/scan. Thus, two independent scans with different collision energies (Ces) were alternatively acquired during the run: a low-energy (LE) scan at a fixed CE of 4 eV, and a high-energy (HE) scan where the CE was ramped from 10 to 40 eV. Argon (99.999%) was used as collision-induced dissociation (CID) gas. Peak identification was accomplished by comparing it with the WATER UNIFI Natural Product Database.

### 2.8. Molecular Docking

The XP (extra precision) option was used to dock an inflexible protein structure with a stretchy ligand (chemical structures used to support target receptors) utilising Schrodinger Glide-v 7.4. A total of 100 poses were created for each docking calculation. Thus, their Van Der Waals (VDW) radius was adjusted as 1.0 with a net atomic charge cut off of 0.25 subunits to soften the potential of nonpolarity elements of protein and ligands, whereas the Van der Waals radius of the other elements was not adjusted. Glide docking uses a conventional cluster-based method to find the best ligand-binding regions in a specified receptor grid field. The ligand with the least XP glide score has the strongest binding affinity for the enzymes [24]. Software was used to study molecular docking for the four target proteins, Bcl-2, Bcl-XL, ERK2, and FAK.

### 2.9. Statistical Analysis

Scratch analysis was figured out by quantifying the cell migration distance using ImageJ software by comparing the distance between untreated and treated cells. Data from multiple scratches within the same test group were analysed using the Analysis of Variance (ANOVA) test to analyse the difference between experimental groups. Data were presented as mean ± S.D. The *p* < 0.05 was considered significant. Other data were analyzed using MS Excel 2010 in Acer Aspire 5 laptop (Acer Inc. New Taipei, Taiwan)

## 3. Results

### 3.1. Extraction Process and Secondary Metabolites Analysis

**Figure 1:**
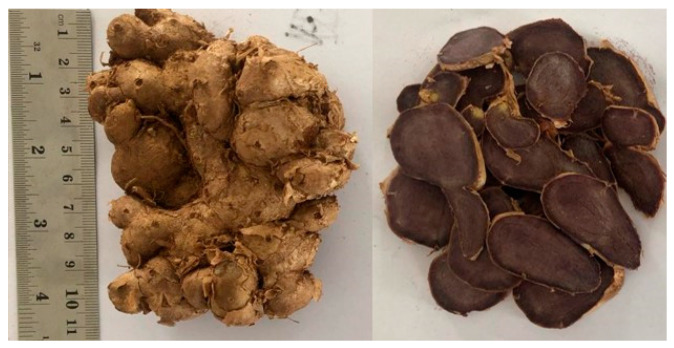
Whole rhizome and cross-sectional appearance of black ginger (*Kaempferia parviflora*) rhizome.

The extraction process from the samples was carried out using the following two different extraction techniques: maceration using 96% ethanol and infusion with distilled water. The KP rhizome used appears dark black and has an odour that is not typical of ginger varieties (Figure 1). Other tests confirmed that the sample used was also black ginger. The ethanol extract yielded 12.98%, while the aqueous extract yielded 12.49%. The two extracted extracts were both black (Table 1).

In addition, secondary metabolite analysis was performed using specific reagents that are dropped on the sample, thereby causing a change in colour or formation of precipitate that can indicate the presence of a class of secondary metabolites. Results of secondary metabolite analysis revealed that both ethanol and water extracts contained secondary metabolites of alkaloids, flavonoids, and polyphenols. Meanwhile, the water extract contained quinone and saponin compounds, whereas the ethanol extract did not (Table 2).

### 3.2. Cytotoxic Activity of Kaempferia parviflora

**Figure 2:**
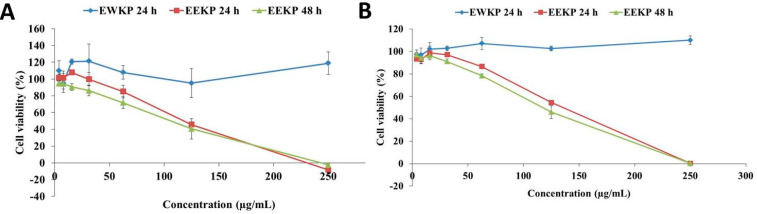
The cytotoxic activity result of EWKP and EEKP against 4T1 breast cancer cell line (**A**) and MCF-7 breast cancer cell line (**B**) in 24 h and 48 h.

MTT assay was used to perform a cytotoxic test on the samples to determine their cytotoxic activity. This cytotoxic test activity of EWKP and EEKP was performed against the breast cancer cell line 4T1 and MCF-7, also in noncancerous cell line NIH-3T3 for 24 h and 48 h. During the 24 h incubation period, results showed that the EWKP did not exhibit any cytotoxic activity both in 4T1 and MCF-7 cell line. Consequently, the EWKP was not tested during the 48 h incubation period (Figure 2). Meanwhile, in NIH-3T3 cytotoxic testing, EWKP and EEKP also had no cytotoxic effect at the highest tested concentration (250 µg/mL). According to the findings of this study, increasing doses of EEKP demonstrated cytotoxic activity at both 24 h and 48 h. Based on the calculations, the IC_50_ of EEKP was 128.33 µg/mL (24 h) and 115.09 µg/mL (48 h) on 4T1 cell line, and 138.43 µg/mL (24 h) and 124.81 µg/mL (48 h) on MCF-7 cell line.

### 3.3. Anti-Migration Activity on 4T1 Breast Cancer Cell Line

**Figure 3:**
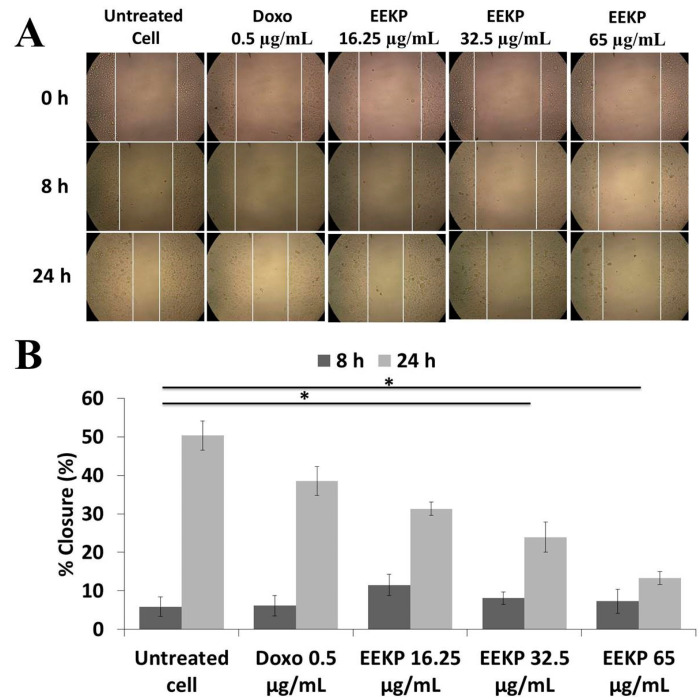
Effect of EEKP and Doxorubicin treatment on cell migration in 4T1 cells (**A**) Morphology of 4T1 cells after being treated with EEKP doses of 16.25 µg/mL, 32.5 µg/mL, and 65 µg/mL and doxorubicin 0.5 µg/mL. Observations were made at 0 h, 8 h, and 24 h using an inverted microscope with 100× magnification. (**B**) Percentage of 4T1 cell closure after 24 h of treatment. The wound area was analyzed using ImageJ software, then the % closure was calculated according to the analytical procedure. (*) indicates a statistically significant difference at the 95% confidence level (*n* = 3).

In addition to a cytotoxic test, to determine the anti-migration activity, the scratch wound healing assay technique was performed as part of this study. Based on the cytotoxic activity findings, only EEKP is continued in this study, because only EEKP has cytotoxic activity. Three nontoxic doses of EEKP were used in the anti-migration study, namely 16.25 µg/mL, 32.5 µg/mL, and 65, µg/mL as well as a positive control of doxorubicin. The experiment lasted for 24 h, with cell movement monitored at 8 h and 24 h. EEKP inhibited cell movement based on the findings. When compared to control cells, the higher the concentration, the greater the inhibition (Figure 3). This anti-migration activity test only uses 4T1 cells because this cell line is a model of highly metastatic breast cancer.

### 3.4. Phytochemical Profiling Using Ultra-High Performance Liquid Chromatography Mass Spectrophotometry (UHPLC-MS)

**Figure 4:**
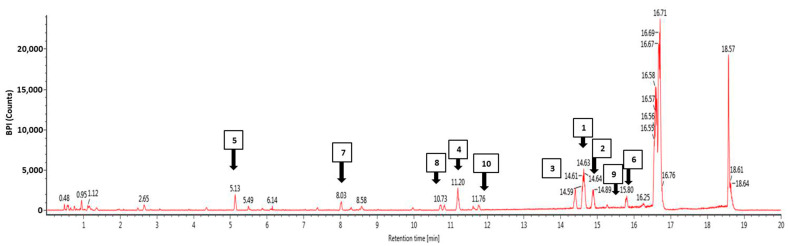
Ultra-High Performance Liquid Chromatography Mass Spectrophotometry (UHPLC-MS) chromatogram, acquired in the positive ion mode, from EEKP where peak labelling represents the compounds identified and listed in Table 3.

The profile of the bioactive compounds found in EEKP was revealed using UHPLC-MS. This study was carried out to investigate previous findings and determine which compound is responsible for the activities. The result showed the EEKP is expected to contain at least 75 bioactive compounds (Figure 4). Then, we chose 10 compounds with the highest response, and four of these were chosen for further in silico molecular docking studies: 5-Hydroxy-7,4′-dimethoxyflavanone (HDMF), 5-Hydro-7,8,2′-trimethoxyflavanone (HTMF), Retusine, and Denbinobin (Table 3 and Figure 4).

### 3.5. In Silico Molecular Docking

**Figure 5:**
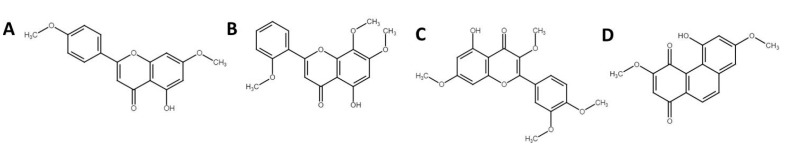
(**A**) 5-Hydroxy-7,4′-dimethoxyflavanone (HDMF), (**B**) 5-Hydro-7,8,2′-trimethoxyflavanone (HTMF), (**C**) Retusine, and (**D**) Denbinobin.

An in silico molecular docking study was performed on the following four compounds: 5-Hydroxy-7,4′-dimethoxyflavanone (HDMF), 5-Hydroxy-7,8,2′-trimethoxyflavanone (HTMF), Retusine, and Denbinobin to predict the bioactive compounds responsible for cytotoxic and antimetastatic activity based on UHPLC-MS prediction results (Figure 5). This in silico study was carried out to predict the compound’s potential binding strength to the chosen receptor. The receptors used in this study are proteins that are involved in the apoptotic pathway (Bcl-2, Bcl-XL) and metastasis (ERK2, FAK). Furthermore, this receptor was chosen based on numerous studies that show KP causes cytotoxicity via intrinsic apoptosis and antimetastatic effects via MMP-9 and MMP-2 production inhibition.

Molecular docking was used to model the possible binding mode of phytochemicals found in KP towards proteins involved in the cytotoxic and metastasis processes. The 3D structures of matrices Bcl-2, Bcl-XL, ERK2, and FAK were obtained from the RCSB protein data bank. Afterwards, the data was prepared by removing crystallographic water and any co-crystallised ligand. Molecular docking simulation was carried out using AutoDock Vina (Vina) with the default settings. The best-docked conformation ranked by vina scoring was used for visual analysis [25]. Pose View, which is accessible through Protein PDB, was used to infer the intermolecular interaction of a protein–ligand complex.

Meanwhile, a molecular docking study was carried out assuming a model where the protein and ligand were considered rigid and flexible, respectively, during the docking procedure [26]. Unfortunately, the sameness in the estimated docking score, the number of H-bonds, and interacting residues are presented in Table 4, Table 5, Table 6 and Table 7. A possible explanation could be the limitation of the docking model to explain how the compounds could arrive at the active site. In all molecular docking experiments, the procedure starts with the ligand located in the site active.

**Figure 6:**
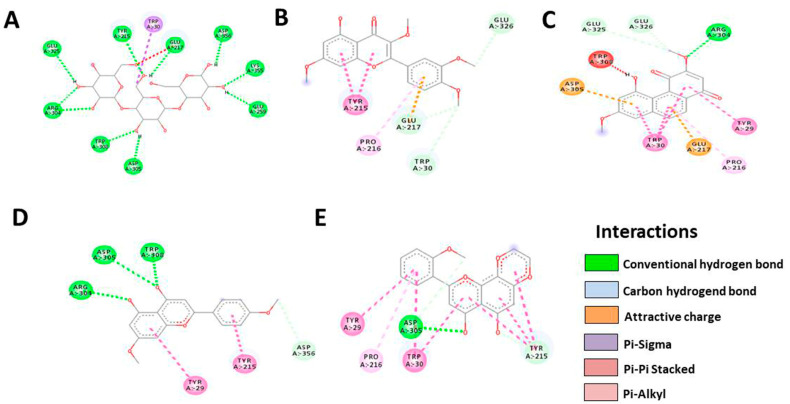
Illustration of bond prediction between native ligands (**A**), Retusine (**B**), Denbinobin (**C**), 5-Hydroxy-7,4′-dimethoxyflavone (HDMF) (**D**), 5-Hydroxy-7,8,2′-trimethoxyflavone (HTMF) (**E**) with Bcl-2 receptor.

**Figure 7:**
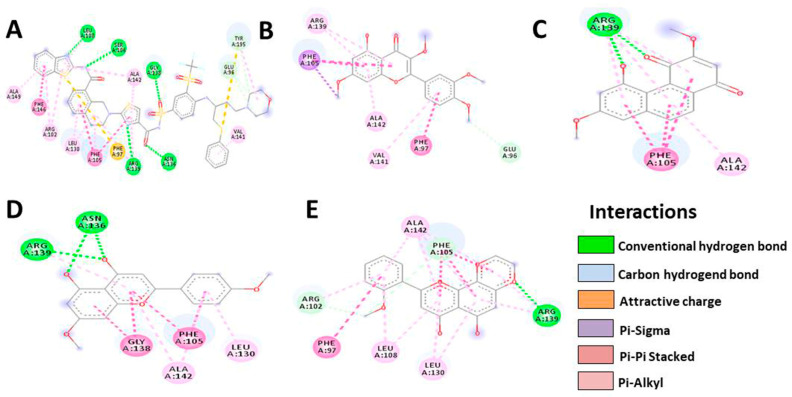
Illustration of bond prediction between native ligands (**A**), Retusine (**B**), Denbinobin (**C**), 5-Hydroxy-7,4′-dimethoxyflavone (HDMF) (**D**), 5-Hydroxy-7,8,2′-trimethoxyflavone (HTMF) (**E**) with Bcl-XL receptor.

**Figure 8:**
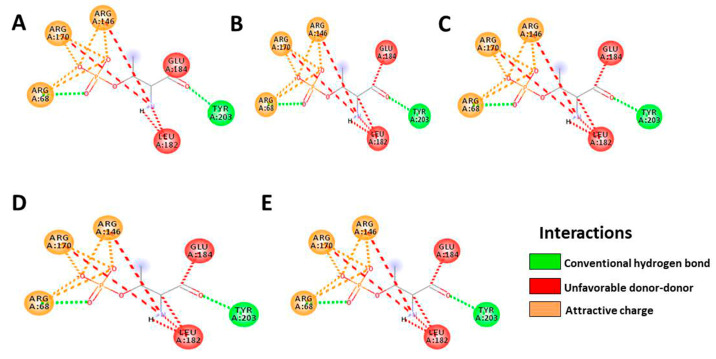
Illustration of bond prediction between native ligands (**A**), Retusine (**B**), Denbinobin (**C**), 5-Hydroxy-7,4′-dimethoxyflavone (HDMF) (**D**), 5-Hydroxy-7,8,2′-trimethoxyflavone (HTMF) (**E**) with ERK2 receptor.

**Figure 9:**
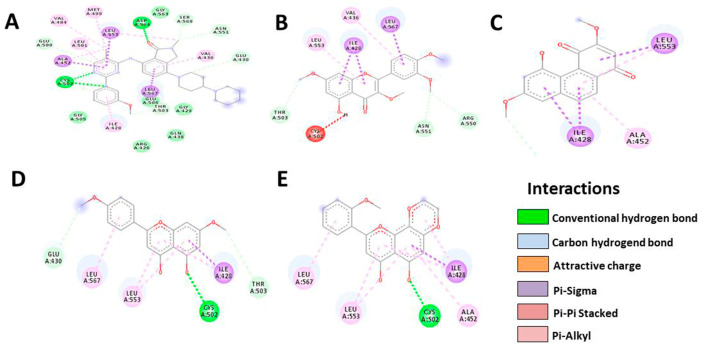
Illustration of bond prediction between native ligands (**A**), Retusine (**B**), Denbinobin (**C**), 5-Hydroxy-7,4′-dimethoxyflavone (HDMF) (**D**), 5-Hydroxy-7,8,2′-trimethoxyflavone (HTMF) (**E**) with FAK receptor.

Based on the results, HTMF has a higher docking score than the native ligand in the Bcl-2 protein, with a value of −9.4, but not in the Bcl-XL protein (Table 4 and Table 5, Figure 6 and Figure 7). Meanwhile, Retusine and Denbinobin are the compounds with the best binding to proteins related to cancer cell migration activity (Table 6 and Table 7, Figure 8 and Figure 9). Consequently, it can be predicted that the compound involved in KP’s cytotoxic activity is 5-Hydro-7,8,2′-trimethoxyflavanone (HTMF), while those involved in antimetastasis are Retusine and Denbinobin.

## 4. Discussion

Breast cancer still has a high incidence and mortality rate today. The treatment of breast cancer also remains a challenge in the health sector [27]. Furthermore, if the breast cancer has migrated and metastasised to other organs, the cancer has progressed to the final stage. This condition has implications for treatment challenges and resistance [28].

Thus, this study primarily aims to identify potential anticancer candidates derived from natural ingredients that can be used in the treatment of metastatic breast cancer. Consequently, black ginger (*Kaempferia parviflora*) has been chosen as a natural ingredient to test on a highly metastatic 4T1 breast cancer cell model, breast cancers cell model MCF-7, and noncancerous cell model NIH-3T3. Even though there has not been much research done on this plant, the literature review suggested that the material used can be developed as an anticancer and anti-migration agent.

This research began with sample extraction using two different extraction procedures and solvents. This extraction process results in two types of extracts: ethanol extract of KP (EEKP) and water extract of KP (EWKP). We tested these two extracts for cytotoxic and anti-migration activity in the highly metastatic 4T1 breast cancer cell model, breast cancers cell model MCF-7, and noncancerous cell model NIH-3T3 in vitro. The result showed that EEKP displayed better cytotoxic activity during the 24 and 48 h treatment periods. Consequently, EEKP was chosen to be studied further.

The cytotoxic activity test results showed that EEKP had cytotoxic activity, with an IC_50_ value of 128.33 g/mL (24 h) and 115.09 g/mL (48 h) in 4T1 cell line and 138.43 µg/mL (24 h) and 124.81 µg/mL (48 h) on MCF-7 cell line. This showed that EEKP has promising anticancer potential. In addition, KP displayed a cytotoxic effect on HeLa, SKOV3, HuCCA-1, RMCCA-1, and T24 cancer cell lines [18,19,21,22,23]. Given that the cytotoxic activity of KP in highly metastatic breast cancer cells has never been reported before, our findings are novel. The water extract from KP had an IC_50_ value that was 19 times lower than the EEKP in the T24 human urinary bladder cancer model [13]. In line with this, it has been shown that EWKP has no cytotoxic activity in 4T1 and MCF-7 cells when concentration was tested.

The next study reported the EEKP’s anti-migration activity. This experiment was carried out using the scratch wound healing assay technique in highly metastasis breast cancer cell line model 4T1. This assay allows for the visualisation of the inhibition of cell movement in the intentional wound area. Three concentration series were chosen for this test based on the previously obtained IC_50_ values. The concentration that was used was nontoxic because scratch wound healing assay studies must use concentrations that allow at least 80% of cells to survive. Our results show that there is a barrier to cell movement, as indicated by EEKP at a dose of 65 g/mL with a 13.53% closure value. This result significantly differs from the % closure value in untreated cells (50.34%). The higher the dose, the greater the inhibitory of EEKP on migration.

Several researchers have also reported KP’s anti-migration and antimetastatic activity, including in HeLa, SKOV3, HuCCA-1, and RMCCA-1 cell lines [20,22,29]. These three studies demonstrated that the ethanol extract of KP inhibited MMP-9 and MMP-2 production. However, the bioactive compound responsible for this activity is not particularly mentioned.

Methoxyflavones, including dimethoxyflavones (DMF), trimethoxyflavones (TMF), tertramethoxyflavones (TMF), and pentamethoxyflavones (PMF) are compounds that provide anticancer activity from KP [30]. These compounds are biomarkers and major components of KP. UHPLC-MS was used to perform phytochemical profiling on EEKP to determine the bioactive compounds responsible for EEKP′s cytotoxic and antimetastatic activity. Based on the WATER UNIFI Natural Product Database, the results of this profiling are predictions of the phytochemical content of the EEKP.

Results of the UHPLC-MS profiling showed the presence of at least 75 bioactive compounds, and we also discovered the presence of methoxyflavones in the form of 5-Hydroxy-7,4′-dimethoxyflavanone (HDMF) at the highest concentration. Besides HDMF, there are previously unknown methoxyflavones, 5-Hydro-7,8,2′-trimethoxyflavanone (HTMF) and two other compounds, Retusine, and Denbinobin. Then, the four compounds were investigated and chosen for in silico molecular docking assays.

Thus, in silico studies were performed to predict which compounds were responsible for the cytotoxic and anti-migration activities. 5-Hydroxy-7,4′-dimethoxyflavanone (HDMF), 5-Hydroxy-7,8,2′-trimethoxyflavanone (HTMF), Retusine, and Denbinobin were selected. Afterwards, these four compounds were tested on several proteins involved in apoptosis (Bcl-2, Bcl-XL) and metastasis (ERK2, FAK).

Various studies on the cytotoxic activity of KP ethanol extract on various cancer cells concluded that KP’s cytotoxic activity was associated with apoptosis induction, particularly in the intrinsic pathway [16]. The intrinsic pathway of apoptosis, also known as the mitochondrial pathway, is where apoptosis occurs primarily as a result of cell-based triggers such as DNA damage, oxidative stress, or metabolic stress. This trigger normally causes the cell to ‘decide’ to die to save other cells and maintain optimal conditions [31].

The Bcl-2 protein family is involved in the regulation of apoptotic on cell [32]. This family consist of pro-apoptotic and anti-apoptotic members [33]. Bcl-2 and Bcl-XL are the example of anti-apoptotic, which block the process of apoptotic by forming complexes with other proteins. Several anticancer studies have been conducted that target Bcl-2 and Bcl-XL as sites of action for the developed anticancer candidates [29,34]. For example, BM-1197, a molecules that targeted Bcl-2 and inhibited the role of this protein on apoptosis, has been developed for the treatment of Diffuse large B cell lymphoma (DLBCL) [35]. This protein is also used as a target of action in the development and search for breast cancer treatments [36].

During the process, the induction ofs BAX and BAK by the BH3 protein has a strong influence on the intrinsic pathway apoptosis process. Other proteins, particularly Bcl-2 and Bcl-XL, have a strong influence on BH3 activity. The presence of these two proteins in their free form can inhibit the process of apoptosis [37]. Thus, inhibition in the form of ‘bonding’ between Bcl-2 and Bcl-XL with a compound will immediately have implications for the initiation of apoptotic events. Our findings show that HTMF binds strongly to the Bcl-2 protein, with a docking value of −9.4. This docking value is greater than that of the native ligand, which is only −9.1. Consequently, we anticipate that HTMF significantly contributes to KP’s cytotoxic activity.

Since migration is a part of cancer cell metastasis, so we used in silico studies of two proteins, ERK2 and FAK. These two proteins are proteins that play a role in cancer cell migration and metastasis. According to the previous study, KP has antimetastatic activity by inhibiting MMP-9 and MMP-2 production. Both of these proteins play critical roles in the invasion of cancer cells. MMP-9 and MMP-2 production can be activated in various ways, one of which is by activating NF-κB via the MEK1 and ERK1/2 pathways [38]. This pathway begins by phosphorylating the MEK1/ERK1/2 cascade, and then ERK1/2 is phosphorylated, hence, it activates NF-κB by removing IkB. Finally, the activated NF-κB/p65/p50 complex moves into the nucleus, thereby causing transcription and Pro-MMP-9 production [39]. Consequently, if a compound can bind to ERK 1/2, MMP-9 production and cell invasion can be inhibited.

**Figure 10:**
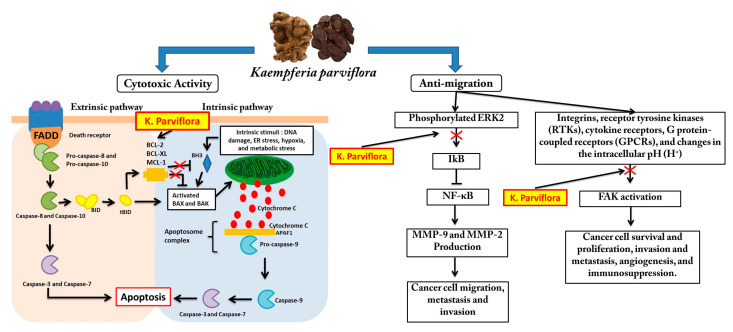
The possible molecular mechanism of Kaempferia parviflora’s cytotoxic and anti-migration activity.

Focal adhesion kinase (FAK), such as MMP-9, is a protein tyrosine kinase that regulates cellular adhesion, motility, proliferation, and survival in various cell types. FAK, which promotes cancer progression and metastasis, is activated and/or overexpressed in advanced cancers. Consequently, FAK has emerged as a potential therapeutic target in cancer [40]. Inhibiting the FAK protein is also thought to aid in cancer healing, particularly in combination treatments. FAK influences the occurrence of cell survival, angiogenesis, and drug resistance in cancer cells in addition to metastasis. In its molecular process, FAK can be activated by presence of Integrins, receptor tyrosine kinases (RTKs), cytokine receptors, G protein-coupled receptors (GPCRs), and changes in the intracellular pH (H^+^) [41]. Based on this, we tested our compound on the FAK protein in addition to the ERK2 protein. We discovered that Retusine and Denbinobin provided a better docking score than the native ligand, namely −8.8 and −9.0 for ERK2, respectively, whereas, in FAK, HTMF provided the best binding value, but not better than the native ligand. As a result of these findings, we predict that HTMF, Retusine, and Denbinobin may play an important role in KP′s anti-migration activity. As a result of our findings, we predict the possible mechanism of KP activity, which is depicted in Figure 10.

5-Hydroxy-7,8,2′-trimethoxyflavone (HTMF) is a methoxyflavone derivative. This compound is the main component of KP and has been shown to be responsible for KP′s biological activity, including anticancer activity [42]. In general, Methoxyflavones are known to be poorly soluble in water, have low lipophilic property, and have low bioavailability [43,44]. This physical character causes methoxyflavones to have a low oral bioavailability of 1% to 4%, with the liver having the highest levels, followed by the kidney. They were also discovered in the lungs, testicles, and brain. As a result, methoxyflavones are primarily eliminated through urine as demethylated, sulfated, and glucuronidated products, and as demethylated metabolites in the faeces [45].

Denbinobin is cytotoxic and antimetastatic in breast and gastric cancer cells [46] and colon cancer cell [47]. This compound is thought to have anti-migration properties via Src-mediated signalling pathways [48]. Meanwhile, Retusine has cytotoxic and antibacterial activity, though this is only based on computer simulations [49]. Thus, we predict that HTMF, Retusine, and Denbinobin play important roles in the cytotoxic and anti-migration activity of KP in breast cancer cells. However, even though our studies have revealed bioactive compounds that play an important role in cytotoxic and anti-migration activities, more research is needed to confirm this. Because our study still relies on predictive studies such as in silico studies, it is preferable if the results are confirmed by other tests such as the transwell migration assay and protein expression assays such as western blotting.

## 5. Conclusions

In conclusion, in vitro studies suggest that KP has anti-migration and cytotoxic activity in breast cancer cells. Based on in silico studies on the Bcl-2, Bcl-XL, ERK2, and FAK receptors, the compounds HTMF, Denbinobin, and Retusine are predicted to contribute to this activity. However, further research is needed to confirm this finding.

## Figures and Tables

**Table 1:** Results of the extraction process from *Kaempferia parviflora*.

	*Kaempferia parviflora*	
	Ethanol	Water
Yield (%)	12.98	12.49
Organoleptic		
Odor	Odorless	Smoky
Color	Black	Black

**Table 2:** The results of phytochemical analysis on ethanol and water extract of *Kaempferia parviflora*.

Type of Secondary Metabolites	*Kaempferia parviflora*		Type of the Test
	Ethanol	Water	
Saponin	−ve	+ve	Saponin test
Tannin	+ve	−ve	Tannin test
Steroid	−ve	+ve	Lieberman Buchard’s test
Alkaloid			
Mayer	+ve	+ve	Mayer’s test
Dragendorf	+ve	+ve	Dragendorf’s test
Buchardat	+ve	−ve	Buchardat’s test
Flavonoid	+ve	+ve	Lead acetate test
Glycosides	−ve	−ve	Keller–Kiliani’s test
Quinone	−ve	+ve	Quinone test
Polyphenol	+ve	+ve	Polyphenol test

**Table 3:** Tentative identification of chemical constituents in the *K. parviflora* ethanol extract.

No	Component Name	Formula	Observed *m*/*z*	Observed RT (min)	Response
1	5-Hydroxy-7,4′-dimethoxyflavanone	C_17_H_14_O_5_	297.0764	14.62	81,895
2	1,3-Dihydroxy-2-ethoxymethyl-anthraquinone	C_17_H_14_O_5_	297.0762	14.88	36,111
3	Retusine	C_19_H_18_O_7_	357.0975	14.4	35,908
4	Denbinobin	C_16_H_12_O_5_	283.0605	11.2	26,614
5	Tectoruside	C_21_H_30_O_13_	489.1611	5.13	24,405
6	5-Hydro-7,8,2′-trimethoxyflavanone	C_18_H_16_O_6_	327.0869	15.79	24,310
7	Acacetin-7-galactoside	C_22_H_22_O_10_	445.1137	8.02	17,504
8	Viscumneoside Ⅵ	C_24_H_26_O_12_	505.1346	10.83	11,985
9	5-Hydro-7,8,2′-trimethoxyflavanone	C_18_H_16_O_6_	327.0869	15.27	10,317
10	3′,5-Dihydroxy-7,4′-dimethoxy flavone	C_17_H_14_O_6_	313.0711	11.77	8933

**Table 4:** The docking score, number of H-bonds, and interacting residues of the selected compounds, Bcl-2.

Bcl-2	Native	Denbinobin	Retusine	HDMF	HTMF
Docking score	−9.1	−8.8	−8.4	−8.1	−9.4
Residue	GLU325	GLU325	GLU326	ARG304	TYR29
	TYR215	GLU326	TRP30	ASP305	PRO216
	TRP30	ARG304	GLU217	TRP308	ASP305
	GLU217	TRP308	PRO216	ASP356	TRP30
	ASP356	ASP305	TYR215	TYR215	TYR215
	LYS355	TRP30		TYR29	
	GLU259	GLU217			
	ASP305	TYR29			
	TRP308	PRO216			
	ARG304				
Number of H-bonds	10	9	5	6	5

**Table 5:** The docking score, number of H-bonds, and interacting residues of the selected compounds, Bcl-XL.

Bcl-XL	Native	Denbinobin	Retusine	HDMF	HTMF
Docking score	−13.4	−8.2	−8.1	−7.9	−8.0
Residue	LEU108	ARG139	PHE105	ARG139	ALA142
	SER106	PHE105	ARG139	ASN136	PHE105
	ALA142	ALA142	ALA142	GLY138	ARG139
	GLY138		VAL141	ALA142	LEU130
	GLU96		PHE97	PHE105	LEU108
	TYR195		GLU96	LEU130	PHE97
	VAL141				ARG102
Number of H-bonds	7	3	6	6	7

**Table 6:** The docking score, number of H-bonds, and interacting residues of the selected compounds, ERK2.

ERK2	Native	Denbinobin	Retusine	HDMF	HTMF
Docking score	−8.7	−9.0	−8.8	−7.5	−7.9
Residue	ARG68	ARG68	ARG68	ARG68	ARG68
	ARG170	ARG170	ARG170	ARG170	ARG170
	ARG146	ARG146	ARG146	ARG146	ARG146
	GLU184	GLU184	GLU184	GLU184	GLU184
	LEU182	LEU182	LEU182	LEU182	LEU182
	TYR203	TYR203	TYR203	TYR203	TYR203
number of H-bonds	6	6	6	6	6

**Table 7:** The docking score, number of H-bonds, and interacting residues, of the selected compounds, FAK.

FAK	Native	Denbinobin	Retusine	HDMF	HTMF
Docking score	−9.8	−7.9	−7.8	−7.3	−8.1
Residue	MET499	LEU553	VAL436	GLU430	LEU567
	VAL484	ALA452	LEU567	LEU567	LEU553
	LEU501	ILE428	ILE428	LEU553	CYS502
	LEU553	THR503	LEU553	CYS502	ALA452
	ASP564		THR503	ILE428	ILE428
	GLY563		CYS502	THR503	
	SER568		ASN551		
	ASN551		ARG550		
Number of H-bonds	8	4	8	6	5

## Data Availability

Data will be made available to any party upon request.
